# Bacterial Chaperone Domain Insertions Convert Human FKBP12 into an Excellent Protein-Folding Catalyst—A Structural and Functional Analysis

**DOI:** 10.3390/molecules29071440

**Published:** 2024-03-23

**Authors:** Gabriel Žoldák, Thomas A. Knappe, Anne-Juliane Geitner, Christian Scholz, Holger Dobbek, Franz X. Schmid, Roman P. Jakob

**Affiliations:** 1 Center for Interdisciplinary Biosciences, Technology and Innovation Park, Pavol Jozef Šafárik University in Košice, 040 11 Kosice, Slovakia; 2 Laboratorium für Biochemie und Bayreuther Zentrum für Molekulare Biowissenschaften, Universität Bayreuth, 95447 Bayreuth, Germany; 3 Roche Diagnostics GmbH, Nonnenwald 2, 82377 Penzberg, Germany; 4 Institut für Biologie, Strukturbiologie/Biochemie, Humboldt-Universität zu Berlin, Unter den Linden 6, 10099 Berlin, Germany; holger.dobbek@biologie.hu-berlin.de; 5 Departement Biozentrum, University of Basel, Spitalstrasse 41, 4056 Basel, Switzerland

**Keywords:** protein folding, chaperone, prolyl isomerase, immunophilin, SlyD, SlpA, FKBP

## Abstract

Many folding enzymes use separate domains for the binding of substrate proteins and for the catalysis of slow folding reactions such as prolyl isomerization. FKBP12 is a small prolyl isomerase without a chaperone domain. Its folding activity is low, but it could be increased by inserting the chaperone domain from the homolog SlyD of *E. coli* near the prolyl isomerase active site. We inserted two other chaperone domains into human FKBP12: the chaperone domain of SlpA from *E. coli*, and the chaperone domain of SlyD from *Thermococcus* sp. Both stabilized FKBP12 and greatly increased its folding activity. The insertion of these chaperone domains had no influence on the FKBP12 and the chaperone domain structure, as revealed by two crystal structures of the chimeric proteins. The relative domain orientations differ in the two crystal structures, presumably representing snapshots of a more open and a more closed conformation. Together with crystal structures from SlyD-like proteins, they suggest a path for how substrate proteins might be transferred from the chaperone domain to the prolyl isomerase domain.

## 1. Introduction

The protein folding of small proteins is typically a very efficient process, and they reach their native conformation within a few milliseconds [1,2,3,4]. The folding of larger proteins is much slower, because it is coupled with slow steps, in particular with prolyl isomerizations [5,6]. Prolyl peptide bonds can exist in trans and cis isomers, and typically the *cis* conformation in the native state complicates folding [7]. Here, the incorrect *trans* isomers predominate in nascent protein chains, and therefore most refolding molecules must undergo *trans*-to-*cis* isomerizations before they can reach the native state [5].

Due to the high energy barrier, prolyl isomerizations are intrinsically slow reactions and nature has evolved enzymes catalyzing peptidyl-prolyl *cis*/*trans* isomerization [8]. Three families of prolyl isomerases are known: the cyclophilins, the parvulins and the FK-506 binding proteins (FKBPs) [9]. Prolyl isomerases can occur as single-domain proteins or as modules of multidomain proteins [10,11]. FKBPs are usually poor protein folding catalysts. Their activity can be increased when the catalytic prolyl isomerase domain is linked to a chaperone domain, as in the trigger factor [12], FKBP22 [13], FkpA [14] or SlyD [15]. Then, the chaperone domain binds folding protein chains and transfers them to the prolyl isomerases.

**Figure 1:**
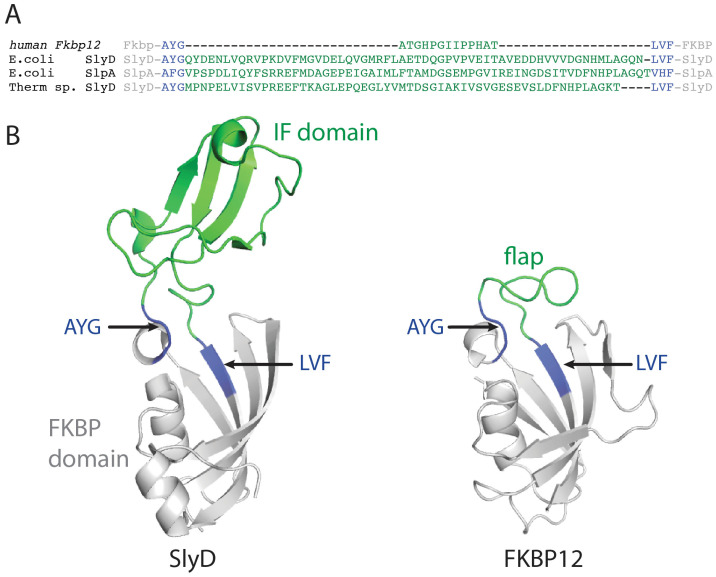
Design of the chimeric proteins: (**A**) Sequence alignment of human FKBP12, *E. coli* SlyD, *E. coli* SlpA, and *Thermococcus thermophilus* FKBP18. The conserved tripeptide units that were used to exchange flap and IF domains are shown in blue, and the flap and insert-in-flap domains are colored green. (**B**) Cartoon representation of E. coli SlyD (EcSlyD; 2K8I.pdb [16], **left**) and human FKBP12 (FKBP12; 1FKF.pdb, **right**). The FKBP domain is shown in grey, and the flap and IF domain are colored green, and the conserved tripeptide units are shown in blue.

The folding enzyme SlyD of *Escherichia coli* consists of a prolyl isomerase domain of the FKBP type and a chaperone domain, which is inserted into a loop of the FKBP domain near the active site (Figure 1A) [16,17]. This loop is called the flap in human FKBP12 (FKBP12), and accordingly, the chaperone domain of SlyD is called the insert-in-flap (IF) domain. The linkers between the two domains are rather long and lead to spatial separation and flexibility between the prolyl isomerase and chaperone domains (Figure 1B) [16].

Human FKBP12 (FKBP12) is a small single-domain prolyl isomerase (Figure 1B) [18] which shows a high sequence specificity towards the residues before the proline [19,20,21]. As it lacks a chaperone domain, it is a weak catalyst of proline-limited protein folding reactions. FKBP12’s folding activity could be strongly increased by inserting the chaperone (IF) domain of its homolog SlyD into the flap loop [22] and additionally reducing the high sequence specificity of FKBP12 [23], creating a generic highly efficient folding enzyme.

In this work, we continued to use the prolyl isomerase FKBP12 and asked whether the chaperone function that is essential for the high activity of prolyl isomerases in protein folding could also be provided by chaperone domains from other SlyD-like folding enzymes. In the designed chimeric FKBP12 variants, two different bacterial chaperone domains were inserted into the flap loop near the catalytic site. These domains were the chaperone domains of the *E. coli* protein SlpA [15,24,25] and of FKBP18 from *Thermococcus* sp. (Figure 1A). By binding to unfolded proteins, they counteract nascent chain aggregation and help in substrate binding. We found that chaperone domain insertion in the chimeric proteins increases the thermodynamic stability, as well as the folding activity of FKBP12. The individual prolyl isomerase and chaperone domain folds were preserved in the designed proteins, as revealed by two crystal structures. The structures in the absence of a substrate represent snapshots of an open and a closed state of the designed enzyme, suggesting similar dynamics as in SlyD proteins. In conclusion, the key to the successful development of highly active catalysts of protein folding lies in determining suitable locations for insertions (e.g., the flap region of FKBP12) and in preserving the native structure of the grafted domain, which is crucial for native inter-domain dynamics.

## 2. Results

### 2.1. The Chimeric Proteins Are Stable and Catalytically Active

In previous experiments, we constructed chimeric proteins in which the flap of human FKBP12 was replaced by the IF domain of *E.coli* SlyD (FKBP12-IF1(SlyD)), yielding an excellent protein folding catalyst [22]. Here, we used the same strategy to interchange the flap of FKBP12 with the IF domains of the SlyD-like proteins SlpA from *E. coli* or FKBP18 from *Thermococcus* sp. SlpA and FKBP18 share the same domain organization as SlyD, but are missing the C-terminal Ni^2+^-ion binding extension. SlpA is suggested to be involved in ribosome biogenesis [24]. FKBP18 shares homology with MtFKBP17 [26] and FKBP26 [27], and is conserved in all *Thermoccoccus* species.

For the enzyme design, we aligned the sequences of FKBP variants with and without an IF domain. In almost all of them, the flap or IF region was surrounded by an Ala-Tyr-Gly section at its amino-terminal side (Ala81-Tyr82-Gly83 in human FKBP12) and a Leu-Xaa-Phe section at its carboxy-terminal side flank (Leu97-Val98-Phe99 in human FKBP12). These sequences are highly conserved whether they lead into a short flap or a long IF domain, and we used them as crossover points in the development of the chimeric prolyl isomerases (Figure 1A).

We aligned the sequences of FKBP variants with and without the IF domain. In most of them, the flap or IF region was flanked by an Ala-Tyr-Gly stretch at its amino-terminal side (A81-Y82-G83 in human FKBP12) and a Leu-Xaa-Phe stretch at its carboxy-terminal side (L97-V98-F99 in human FKBP12). These sequences were well conserved, irrespective of whether they lead into a short flap or into a long IF domain, and we used them as the cross-over points in the construction of the chimeric proteins (Figure 1A).

The designed variants of human FKBP12, FKBP12-IF(SlpA) and FKBP12-IF(FKBP18) contain a hexahistidine tag and C22A substitution [28]. To measure the thermodynamic stability of the protein, urea- and GdmCl-induced unfolding transitions were recorded, using the fluorescence of the single Trp residue (W59) of FKBP12 that strongly increases upon unfolding [29]. The insertion of the IF domain of SlpA or *Thermococcus* sp. SlyD into FKBP12 increased the midpoint of GdmCl-induced unfolding and stabilized it by about 5 and 8 kJ mol^−1^, respectively (Table 1). This demonstrates that the two chain connections between the FKBP domain and the IF domain fit properly.

The proteins SlpA and FKBP18 naturally contain IF domains. The excision of the IF domain decreases the midpoint of the GdmCl-induced unfolding transition from 1.7 M to 1.4 M GdmCl (Table 1) [25], which corresponds to a destabilization by 5.8 kJ mol^−1^. In the case of FKBP18, the excision of the IF domain increases the midpoint of the GdmCl-induced unfolding transition from 3.6 M to 3.8 M GdmCl, but the cooperativity is decreased from 10.0 to 7.4 kJ mol^−1^ M^−1^, corresponding to a decrease in stability by 7.8 kJ mol^−1^, in line with experiments of SlyD from *T. thermophilus* [17]. The destabilization of SlpA and FKBP18 upon IF domain removal (5.8 to 7.8 kJ mol^−1^) is in the same range as the stability gain upon the insertion of their IF domains in FKBP12, confirming the good protein design.

**Figure 2:**
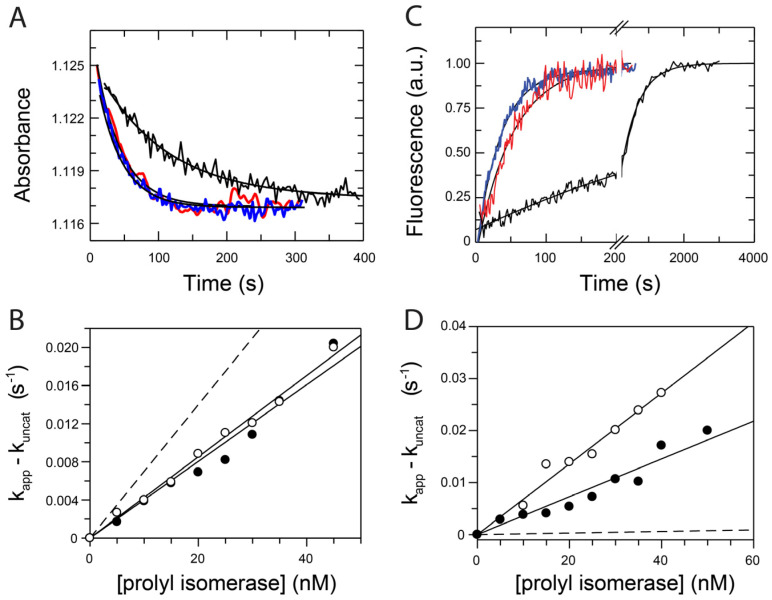
Characterization of the enzymatic activities of the chimeric enzymes. (**A**) *Cis*-*trans* isomerization kinetics of 1.5 μM Suc-Ala-Leu-Pro-Phe-*p*Na followed by an absorbance change at 330 nm, without enzyme (black), and with 40 nM FKBP12-IF(FKBP18) (blue) and 40 nM FKBP12-IF(SlpA) (red). The black curves indicate the fit to the data. (**B**) Catalytic efficiencies of FKBP12-IF(SlpA) (filled circles) and FKBP12-IF(FKBP18) (open circles) in the protease-free assay with tetrapeptide Suc-Ala-Leu-Pro-Phe-p-nitroanilide. For comparison, data for FKBP12 are shown as a dashed line. Solid lines were obtained from linear regression analysis. (**C**) Time course of the refolding of 0.1 μM RCM-T1 in the absence (black curve) and in the presence of 40 nM FKBP12-IF(FKBP18) (blue curve) and 40 nM FKBP12-IF(SlpA) (red curve). Refolding was initiated by the solvent jump from 0.1 M Tris/HCl, pH 8.0 (at 15 °C) to 2 M NaCl, 0.1 M Tris/HCl, pH 8.0 (at 15 °C) at 15 °C. Solid curves were obtained from the non-linear regression analysis of the single exponential reaction. (**D**) Catalytic efficiencies of FKBP12-IF(FKBP18) (open circles) and FKBP12-IF(SlpA) (filled circles). The solid line was obtained from linear regression analysis. The slope yields the catalytic efficiencies of the enzymes for the protein substrate. The dashed line represents the catalytic efficiency of FKBP12. The *k*_cat_/*K*_m_ values derived from the slopes are given in Table 2.

The tetrapeptide Suc-Ala-Leu-Pro-Phe-4-nitroanilide was used in a protease-free prolyl isomerase assay to determine the prolyl isomerase activity of the variants [30]. The insertion of IF domains did not change the catalytic activity of the prolyl isomerase active site towards a short peptide (Figure 2A,B). This activity was not altered by the excision of the IF domain from SlpA or FKBP18 or by the insertion of IF domains into FKBP12 (Table 2).

### 2.2. Chimeric Fusion Proteins Catalyze the Protein Refolding Very Well

Next, we determined the influence of the IF chaperone domain on the catalytic activity of the chimeric proteins as catalysts of proline-limited protein folding (protein folding activity). As a substrate protein, we used the reduced and carboxymethylated form of the S54G/P55N variant of ribonuclease T_1_ (RCM-T1) [31]. In the native state, RCM-T1 contains a single *cis* prolyl bond (Tyr38-Pro39). In the absence of salt, the protein is unfolded and refolding starts with a jump to 2.0 M NaCl. Overall, 85% of the unfolded RCM-T1 refolds in a monophasic reaction, which is limited by the slow *trans*-to-*cis* isomerization at Pro39. The uncatalyzed refolding of RCM-T1 shows a time constant of 530 s (at 15 °C, pH 8.0). FKBP12-IF(SlpA) and FKBP12-IF(FKBP18) are excellent catalysts of RCM-T1 folding. In the presence of only 10 nM of each enzyme, slow refolding was enhanced approximately fivefold (Figure 2C,D), comparable to the natural folding enzymes SlyD and FKBP18 (Table 2).

### 2.3. Chimeric Fusion Proteins Are Good Chaperones

**Figure 3:**
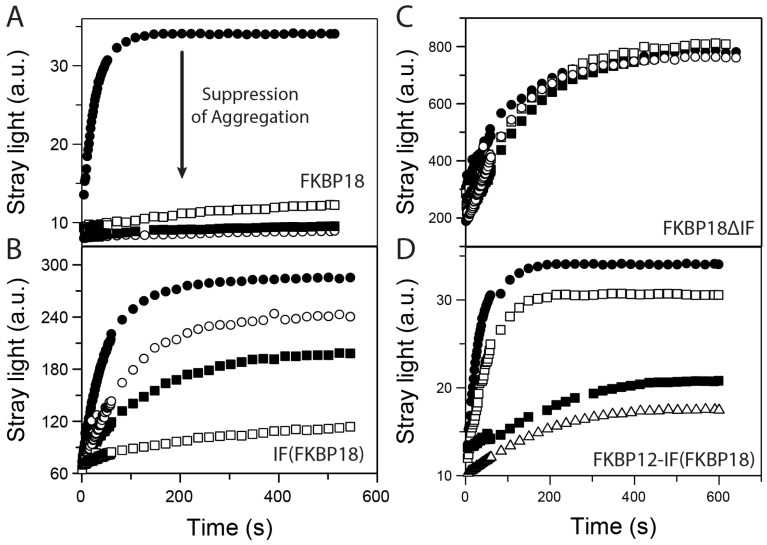
Chaperone function of the FKBP18 and FKBP12-IF(FKBP18) variants. (**A**) Aggregation of citrate synthase at 25 °C in the absence (filled circle, ●) and in the presence of 1 µM (open squares, □), 2 µM (filled squares, ■) and 3 μM (open circles, ⚬) of FKBP18. (**B**,**C**) Aggregation of denatured citrate synthase in the absence (filled circle, ●) and in the presence of 6 µM (open squares, □), 12 µM (filled squares, ■) and 24 μM (open circles, ⚬) of FKBP(FKBP18) (**B**) and IF(FKBP18) (**C**). (**D**) Aggregation of citrate synthase at 25 °C in the absence (filled circle, ●) and in the presence of 1 µM (open squares, □), 2 µM (filled squares, ■) and 3 μM (open triangles, Δ) FKBP12-IF(FKBP18).Unfolded citrate synthase in 6 M GdmCl was diluted to a final protein concentration of 0.15 μM in 50 mM Tris–HCl (pH 8.0), 30 mM GdmCl and 0.1 mM DTE. The light-scattering increase upon aggregation was monitored at 360 nm.

Using the citrate synthase assay, the chaperone activities of the SlpA, FKBP18 and FKBP12 variants were analyzed [32]. Unfolded citrate synthase in 6.0 M guanidinium chloride (GdmCl) aggregated upon dilution to 30 mM GdmCl, which was accompanied by a light-scattering increase. FKBP18 is an efficient chaperone. FKBP18 at 1 μM led to a partial and 3 µM to a complete inhibition of citrate synthase aggregation (Figure 3A), similar to SlyD (Table 2) [33]. The isolated IF domain of FKBP18 also acts as a chaperone, but with reduced efficiency compared to full-length FKBP18 (Figure 3B). FKBP18ΔIF did not slow down citrate synthase aggregation, even when present at a 150-fold excess (Figure 3C). SlpA suppressed citrate synthase aggregation well, whereas SlpAΔIF only exhibited an insignificant chaperone activity (Table 2), similar to deletion mutants of MtFKBP17 and SlyD, which lack the IF-domain [15,22,26]. FKBP12 with the IF domain of FKBP18 [FKBP12-IF(FKBP18)], which catalyzes protein folding as well as FKBP18, is, however, a less active chaperone; 3 μM FKBP12-IF(FKBP18) barely suppressed the citrate synthase aggregation, and 24 μM only partially inhibited it (Figure 3D). All three FKBP12-IF chimeric proteins tested exhibited comparable chaperone activities, but significantly lower than the respective activities of SlyD or FKBP18.

### 2.4. FKBP12-IF(SlyD) and FKBP12-IF(SlpA) Crystal Structures

**Figure 4:**
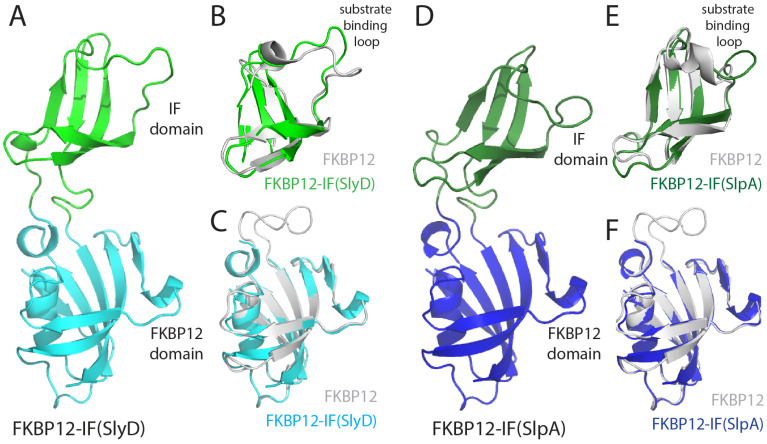
Crystal structures of FKBP12-IF(SlyD) and FKBP12-IF(SlpA) in comparison to their parent proteins FKBP12, SlyD and SlpA. (**A**) Crystal structure of FKBP12-IF(SlyD). The FKBP12 and IF domains are colored cyan and green, respectively. (**B**,**C**) Superposition of the IF domain and FKBP domain of FKBP12-IF(SlyD) (gray) to their parent proteins, (**B**) the IF domain of SlyD (PDB ID: 2K8I, gray) and (**C**) FKBP12 (PDB ID: 1FKF, gray). (**D**) Crystal structure of FKBP12-IF(SlpA). The FKBP12 and IF domains are colored in blue, and dark green, respectively. (**E**) Superposition of the IF domain and FKBP domain of FKBP12-IF(SlpA) (gray), and (**F**) superposition of the FKB12 domain of SlpA (PDB ID: 4DT4, gray) and FKBP12 (PDB ID: 1FKF, gray).

To understand the structural consequences of IF domain insertion in FKBP12, we crystallized FKBP12-IF(SlpA) and FKBP12-IF(SlyD), the properties of which have been analyzed previously [22]. The crystal structures were solved by molecular replacement with the structure of FKBP12 (pdb: 1FKF) [18] as a search model and refined at resolutions of 2.0 Å and 1.9 Å to *R*_work_/*R*_free_ values of 20.5/24.0% and 20.3/23.9%, respectively (Table 3), including one molecule in the asymmetric unit. All residues except the C-terminal hexa-histidine tag could be modeled. FKBP12-IF(SlpA) and FKBP12-IF(SlyD) are composed of the FKBP domain, including the canonical β-strands 2–5 and α-helix 1 motif, and the IF domain, which is characterized by an incomplete β-barrel structure (Figure 4A). In FKBP12-IF(SlyD), the FKBP12 and IF domains superpose closely to their parental proteins FKBP12 and SlyD with overall root mean square deviations (C_α_ r.m.s.d) of 0.35 Å and 1.5 Å, respectively (Figure 4B,C). Minor differences were observed for the substrate binding loop in the IF domain (Figure 4B). In fact, this region shows variable conformations and high flexibility in different crystal and NMR structures of *T. thermophilus* SlyD [16,17,34]. Similarly, the FKBP12 and IF domains in FKBP12-IF(SlpA) (Figure 4D) superpose closely to their parental proteins, with 0.7 Å (FKBP domain) and 1.3 Å (IF domain) r. m. s. deviations (Figure 4E,F), demonstrating that the domain folds are closely retained in both FKBP12-IF(SlpA) and FKBP12-IF(SlyD).

**Figure 5:**
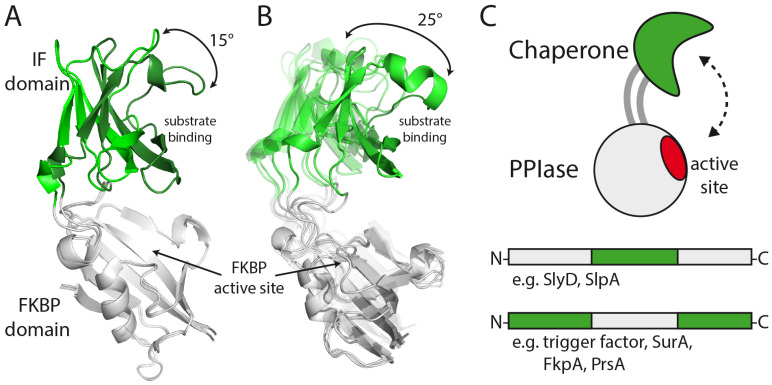
Crystallographic snapshots of functional states of SlyD-like proteins. (**A**) FKBP12-IF(SlyD) is shown in grey (FKBP12 domain) and green (IF domain), superimposed on FKBP12-IF(SlpA) (FKBP domain: grey; IF domain: forest) based on the FKBP12 domain. (**B**) Superimposition of SlyD-like proteins in the open and closed conformation: *T. thermophilus* SlyD (3LUO.pdb), FKBP12-IF(SlyD) (5I7P.pdb), FKBP12-IF(SlpA) (5I7Q.pdb), and *E. coli* SlpA (PDB ID: 4DT4.pdb) based on the FKBP12 domain. The IF domain positions follow a trajectory towards the substrate binding site of the FKBP domain, animated in the Supplementary Movie S1. (**C**) Schematic model of folding helper proteins consisting of prolyl isomerase and the chaperone domain. In nature, the insertion of the chaperone domain in a prolyl isomerase domain (e.g., SlyD-like proteins) or vice versa, the insertion of a prolyl isomerase in a chaperone domain (e.g., trigger factor, SurA, PrsA), are found, and likely share similar protein dynamics.

However, the orientations of the FKBP and IF domains relative to each other differ between the two crystal structures. FKBP12-IF(SlpA) represents a more closed conformation, whereas FKBP12-IF(SlyD) exhibits a more open conformation (Figure 5A). Both conformations were also observed in a normal mode analysis, which was used to study protein dynamics with large amplitude, suggesting that both structures represent snapshots of the native proteins [35,36]. This finding is in line with several crystal structures of *Thermus thermophilus* SlyD and SlpA (Figure 5B), where different orientations of the FKBP12 and IF domains were found, as well, presumably determined by varying crystal packing interactions [17,24]. The chimeric proteins apparently show domain orientations and domain dynamics as the native hosts, which explains their equally high efficiency as catalysts in the protein folding experiments (Table 2). The superposition of crystal structures of *Thermus thermophilus* SlyD, SlpA and the two chimeric proteins based on the FKBP domain (Figure 5B) suggest a trajectory for an IF domain opening and closing motion of SlyD-like proteins, already sampled in the absence of substrates (Supplementary Movie S1).

## 3. Discussion

### 3.1. Domain Exchange between FKBP12 and SlyD-like Proteins

FKBP12, FKBP18 and SlpA share a low 13% sequence identity between their FKBP domains. Nevertheless, the flap of FKBP12 (19 residues) and the IF domain (~65 residues) could be exchanged between the proteins without affecting the catalytic prolyl isomerase activities towards tetrapeptide substrates (Table 2). This corroborates previous experiments in which the IF domain of SlyD could be successfully inserted into the flap of FKBP12 [22].

The thermodynamic stability of the chimeric proteins was not impaired either. The insertion of the IF domain of SlpA or of FKBP18 into FKBP12 even increased the stability of FKBP12. This is remarkable, as the insertion of a foreign domain into the loop of another protein is intrinsically destabilizing. This may be because local chain contacts are converted into nonlocal ones (which is entropically unfavorable) and because domain grafting often leads to structural mismatches [37,38], as found for the insertion of the chaperone domain of SlyD proteins into the flap loop of FKBP12 [22]. This tolerance to domain exchange might be explained by the intrinsically higher stability of the IF domains of SlpA and FKBP18 than the IF domain of SlyD. Additionally, despite very low overall sequence identity, SlpA, FKBP18 and FKBP12, show similar sequences in the chain segments connecting the FKBP domain with the IF domain or the flap (Figure 1A). Other successful domain insertion experiments used T4-Lysozyme ([39,40]), but often required a more extensive screening of functional protein variants.

In the case of SlyD, we showed previously that the guest IF domain behaves as a “servant” that obeys the “master” FKBP domain. It folds only when the FKBP domain is folded and follows this domain when it unfolds [41]. In contrast, the IF domains of SlpA and FKBP18 are thermodynamically stable in isolation (Table 1). Hence, the master–servant relationship which holds for SlyD is broken and, as a consequence, the IF domains contribute to the overall folding stability of the master FKBP domain.

### 3.2. The Folding Efficiency of FKBP12 Is Improved by Chaperone Domains

When the IF domains of SlpA or FKBP18 are inserted into FKBP12, its folding activity increases strongly to a level that is much higher than the folding activity of the IF domain donor (in the case of SlpA), or is similarly high (in the case of FKBP18) (Table 2). Therefore, the presence of the IF domain is required for high catalytic efficiency in proline-limited folding. But the high catalytic protein folding efficiency does not correlate with its performance in the citrate synthase chaperone assay; FKBP12-IF(FKBP18) has a similar folding activity to FKBP18, but a lower efficiency in the citrate synthase aggregation. FKBP12-IF(SlpA) has a 100-fold higher protein folding efficiency than SlpA but performs similarly well in the chaperone assay.

### 3.3. Structure and Large-Scale Dynamics of Chimeric Proteins

The structure determination of two chimeric proteins revealed that IF domain insertion does not affect the FKBP12 and IF domains. Both domains show close similarities to their parental proteins and are the sum of the two parts. Even the linker sequences leading from the IF domain to the FKBP12 domain are very similar to their native proteins SlyD and SlpA (Figure 4).

In SlyD and SlyD-like proteins, fast dynamics between the IF and the FKBP domains exist to facilitate the efficient catalysis of prolyl *cis*/*trans* isomerization [42]. These dynamics are already present in the absence of the substrate and are correlated with motions on different timescales [43]. Large-scale motions of the FKBP and IF domains of SlyD relative to each other were also confirmed by single molecule FRET experiments [44]. A broad distribution of distances was observed with two maxima that were assigned to a rapid exchange between an open and a closed conformation. Interestingly, relative populations of these conformations and their dynamics did not depend on the presence of the bound substrate, suggesting that the domain dynamics are driven only by thermal fluctuations. In other words, SlyD is a system with passive dynamics and, hence, does not rely on a highly sophisticated directed signal transduction path as found, for example, for the ATP-fueled two-domain DnaK chaperone [45]. Along the DnaK signal transduction path, a single amino acid exchange completely abolished the communication between domains, thus leading to a loss-of-function phenotype. SlyD, on the other hand, appears to be a “passive” system. Diverse chaperone domains could be introduced in unrelated FKBP12 domains, and the resulting chimeric variants displayed a high folding activity, comparable to the natural folding enzyme SlyD.

The two new crystal structures determined here revealed a closed conformation for FKBP12-IF(SlpA) and an open conformation for FKBP12-IF1(SlyD), which possibly represent snapshots of conformational sub-states (Figure 5A). Both crystal structures are on a trajectory with other conformational states found for *Thermus thermophilus* SlyD and SlpA, also suggesting that in the chimeric proteins, large-scale motions of FKBP and IF domain might occur with a similar magnitude (Supplementary Movie S1). The intrinsic passive dynamics of SlyD-like proteins are not changed in the chimeric proteins because the insertion points and the linker sequences (transferred together with the IF domains) are maintained in the chimeras (Figure 1A).

The insertion of a chaperone domain in a prolyl isomerase fold, as in SlyD or SlpA, or alternatively the insertion of a prolyl isomerase domain in a chaperone domain, as in the trigger factor, PrsA, FkpA or SurA, seems to be a common concept in folding enzymes [5]. They share the same functional principle: the substrate is bound by one or more chaperone domains and then transferred to a prolyl isomerase domain. Thus, such a directed but passive chaperone to prolyl isomerase domain motion, as found for SlyD-like proteins, might be a general feature and probably improves the efficiency of the substrate transfer. Domain insertions are found in many structurally and functionally unrelated enzymes [46,47,48,49,50], and passive motion might be used here as well to support substrate transfer and catalysis.

## 4. Materials and Methods

The protein variants were produced and purified as described [40]. (S54G/P55N) RNase T_1_ was purified, reduced and carboxymethylated by the procedure used for wild-type RNase T_1_ [31].

### 4.1. GdmCl and Urea-Induced Unfolding Transitions

Fluorescence measurements were performed with a Jasco (Tokyo, Japan) FP6500 fluorescence spectrophotometer. Unfolding transitions were measured by the change in tyrosine fluorescence at 310 nm (5 nm bandwidth) after excitation at 280 nm (3 nm bandwidth) in 0.1 M K-phosphate and 1 mM EDTA (pH 7.5) at 15 °C. The experimental data were analyzed according to a two-state model by assuming a linear dependence of fluorescence emission on urea concentration. A nonlinear least-squares fit of the experimental data was used to obtain the Gibbs free energy of denaturation Δ*G* as a function of denaturant concentration [51].

### 4.2. Prolyl Isomerase and Chaperone Activity Assays

The prolyl isomerase activities were measured by a protease-free fluorescence assay [30] and the folding experiments with RCM-T1 were performed as described [31]. For the chaperone activity assay, citrate synthase (30 µM) was unfolded in 50 mM Tris–HCl (pH 8.0), 20 mM dithioerythritol (DTE), 6.0 M GdmCl for 1 h and then diluted 200-fold, in the presence of various concentrations of prolyl isomerase at 25 °C [4]. The increase in light scattering at 360 nm was used to monitor spontaneous aggregation.

### 4.3. Protein Crystallization and Structure Determination

FKBP12-IF(SlyD) and FKBP12-IF(SlpA) were crystallized by hanging-drop vapor diffusion at 20 °C. FKBP12-IF(SlyD) crystallized in 20% *PEG3350*, 0.1 M MgCl_2_, 0.1 M Hepes, pH 7.5, and crystals were flash frozen in liquid nitrogen after the addition of 20% glycerol. Crystals of FKBP12-IF(SlpA) (500 μL) were obtained in 25% *PEG1500*, 10% *isopropanol*, 0.1 M CaCl_2_, 0.1 M MES, pH 6.5 and flash frozen after increasing the isopropanol concentration to 20%. Crystals were measured using an in-house X-ray facility consisting of a rotating Cu anode (Nonius FR 591, Bruker AXS, Karlsruhe, Germany) coupled to an image plate detector (mar345dtb, Marresearch, Hamburg, Germany). The data sets were processed and scaled using XDS [52,53]. FKBP12-IF(SlyD) crystals belonging to the space group *P*_4_2_1_2 with cell dimensions *a* = 60.2 Å, *b* = 60.2 Å and *c* = 120.5 Å, with one molecule per asymmetric unit. The FKBP12-IF(SlpA) crystals belonged to space group *P*22_1_2, with cell dimensions *a* = 46.7 Å, *b* = 54.8 Å and *c* = 63.6 Å, and contained one molecule per asymmetric unit. The structures were determined by molecular replacement with the FKBP12 structure as the search model (PDB-ID: 1fkk; [54]) using the program Phaser [55]. Model building and structure refinement were performed with Coot [56] and PHENIX [57], respectively, and are summarized in Table 3 The atomic coordinates for FKBP12-IF(SlyD) and FKBP12-IF(SlpA) have been deposited in the RCSB Protein Data Bank and are available under the accession code 5I7P and 5I7Q, respectively.

## Figures and Tables

**Table 1:** Effect of the IF domain on the stabilities of human FKBP12, SlyD and SlpA.

Prolyl Isomerase	[D]_M_ (M)	*m* (kJ mol^−1^ M^−1^)	Δ*G* (kJ mol^−1^)	Reference
FKBP12	2.6	7.8	20	urea
SlyD	2.6	6.3	16.2	urea ^a^
SlyD ΔIF	3.2	4.8	15.2	urea ^a^
IF(SlyD)	-	-	unfolded	urea ^a^
FKBP12-IF(SlyD)	1.9	8.1	15.2	urea ^a^
SlpA	1.7	11.6	19.9	GdmCl
SlpaΔIF	1.4	9.8	14.1	GdmCl
IF(SlpA)	1.2	8.2	10.1	GdmCl
FKBP12-IF(SlpA)	1.3	20.0	25.0	GdmCl
FKBP18	3.6	10.0	36.0	GdmCl
FKBP18ΔIF	3.8	7.4	28.3	GdmCl
IF(FKBP18)	0.6	10.8	6.3	GdmCl
FKBP12-IF(FKBP18)	5.0	5.6	27.8	urea

Due to the large difference in stability, the data were derived under different conditions. The stabilities of FKBP12 and *E. coli* SlyD(1-165) were measured at 10 °C, pH 7.0 from urea-induced unfolding transitions [22], and the stabilities of SlyD from *Thermococcus* sp. and *E. coli* SlpA were derived at 15 °C, pH 7.5 from GdmCl-induced unfolding transitions. The transition midpoints ([D]_M_), the cooperativity values (*m*) and unfolding free energies (Δ*G*) are given. ^a^ Thermodynamic stability values of FKBP12, FKBP12-IF(SlyD) and SlyD variants are taken from Ref. [22].

**Table 2:** Catalytic efficiencies of wild-type and chimeric proteins towards the *cis*-to-*trans* isomerization of the proline containing tetrapeptide and the refolding of RCM-T1 and chaperone activity in a citrate synthase-based aggregation assay.

Protein	Tetrapeptide *^a^* *k*_cat_/*K*_M_ (M^−1^·s^−1^)	RCM-T1 *^b^* *k*_cat_/*K*_M_ (M^−1^·s^−1^)	Chaperone Activity *^d^*
FKBP12 *^c^*	0.7 × 10^6^	1.50 × 10^4^	−
SlyD *^c^*	0.25 × 10^6^	0.82 × 10^6^	+++
SlyD ΔIF *^c^*	0.23 × 10^6^	no activity	−
IF(SlyD) *^c^*	no activity	no activity	−
FKBP12-IF(SlyD) *^c^*	0.71 × 10^6^	2.9 × 10^6^	++
SlpA	0.01 × 10^6^	2.48 × 10^3^	++
SlpaΔIF	0.01 × 10^6^	2.50 × 10^3^	−
IF(SlpA)	no activity	no activity	+
FKBP12-IF(SlpA)	0.48 × 10^6^	0.36 × 10^6^	++
FKBP18	0.21 × 10^6^	0.68 × 10^6^	+++
FKBP18ΔIF	0.30 × 10^6^	2.0 × 10^3^	−
IF(FKBP18)	no activity	no activity	++
FKBP12-IF(FKBP18)	0.42 × 10^6^	0.66 × 10^6^	++

*^a^* Protease-free activity assay with the tetrapeptide succinyl-Ala-Leu-Pro-Phe-p-nitroanilide at 15 °C in 35 mM Hepes/NaOH, pH 7.8 and 1 μM bovine serum albumin using absorbance changes at 330 nm. *^b^* Enzyme activities were determined during the refolding of RCM-T1 at 15 °C in 2.0 M NaCl, 0.1 M Tris/HCl, pH 8.0 using a change in tryptophan fluorescence. *^c^* Prolyl isomerase activity data of FKBP12, FKBP12-IF(SlyD) and *E. coli* SlyD variants are taken from Ref. [22]. *^d^* Citrate synthase aggregation at 25 °C, as shown in Figure 3, (−) to (+++) indicates the range of measured activity, (−) no activity, (+) low activity, (++) medium activity, and (+++) high chaperone activity.

**Table 3:** Statistics on diffraction data and structure refinement of the FKBP12-IF(SlyD) and FKBP12-IF(SlpA).

Data Set	FKBP12-IF(SlyD)	FKBP12-IF(SlpA)
PDB ID	5I7P	5I7Q
Wavelength (Å)	1.5418	1.5418
Space group	*P* 4_1_2_1_2	*P* 2_1_2_1_2_1_
Unit cell (Å, °)	60.2 60.2 120.5 90 90 90	54.8 63.6 46.7 90 90 90
Resolution (Å)	30–2.0 (2.1–2.0) *	35.0–1.9 (2.0–1.9) *
Total reflections	92,295	45,572
Unique reflections	15,492	12,984
Multiplicity	5.9 (5.8)	3.5 (3.6)
Completeness (%)	99.1 (99.2)	97.2 (99.7)
Mean I/sigma(I)	22.0 (2.6)	14.3 (3.0)
Wilson B-factor (Å^2^)	29.4	23.5
R-merge	0.061 (0.560)	0.144 (0.527)
CC_1/2_	99.9 (92.0)	99.9 (82.2)
R-work	0.206 (0.237)	0.203 (0.216)
R-free	0.250 (0.295)	0.238 (0.258)
Number of atoms	2566	2716
Macromolecules	1205	1238
Water	175	240
Protein residues	153	156
RMS(bonds) (Å)	0.005	0.006
RMS(angles) (°)	0.95	1.00
Ramachandran favored (%)	99	98
Ramachandran outliers (%)	0	0
Clashscore	2.1	2.0
Average B-factor (Å^2^)	36.8	18.6
Macromolecules	36.1	17.1
Solvent	41.8	26.6

* Values in parentheses are for highest resolution shell.

## Data Availability

Data are contained within the article and Supplementary Materials.

## Supplementary Materials

The following supporting information can be downloaded at: https://www.mdpi.com/article/10.3390/molecules29071440/s1, Supplementary Movie S1. Structures of the FKBP12 domain are aligned and animated from *Thermus thermophilus* SlyD over PDB ID: 3LUO, 5I7Q, 5I7P, 4DT4. The IF domain positions differ by a rotation of 25° around the common FLAP region in the FKBP12 domain.

## Abbreviations

Abz, aminobenzoyl; pNA, para-nitroanilide; RCM-T1, reduced and carboxymethylated form of the S54G/P55N variant of RNase T1; FKBP, FK506 binding protein; CS, citratesynthase; GdmCl, guanidinium chloride.

## References

[1] Schindler T., Herrler M., Marahiel M.A., Schmid F.X. (1995). Extremely rapid folding in the absence of intermediates: The cold-shock protein from Bacillus subtilis. Nat. Struct. Biol..

[2] Ferguson N., Fersht A.R. (2003). Early events in protein folding. Curr. Opin. Struct. Biol..

[3] Huang G.S., Oas T.G. (1995). Submillisecond folding of monomeric lambda repressor. Proc. Natl. Acad. Sci. USA.

[4] Mayor U., Johnson C.M., Daggett V., Fersht A.R. (2000). Protein folding and unfolding in microseconds to nanoseconds by experiment and simulation. Proc. Natl. Acad. Sci. USA.

[5] Schmidpeter P.A., Schmid F.X. (2015). Prolyl isomerization and its catalysis in protein folding and protein function. J. Mol. Biol..

[6] Schiene-Fischer C., Aumüller T., Fischer G. (2013). Peptide bond cis/trans isomerases: A biocatalysis perspective of conformational dynamics in proteins. Top. Curr. Chem..

[7] Stewart D.E., Sarkar A., Wampler J.E. (1990). Occurrence and role of cis peptide bonds in protein structures. J. Mol. Biol..

[8] Schmid F.X. (2002). Prolyl isomerases. Adv. Protein Chem..

[9] Fischer G. (1994). Peptidyl-prolyl cis/trans isomerases and their effectors. Angew. Chem. Int. Ed. Engl..

[10] Schiene-Fischer C. (2015). Multidomain peptidyl prolyl cis/trans Isomerases. Biochim. Biophys. Acta.

[11] Galat A. (2003). Peptidylprolyl cis/trans isomerases (immunophilins): Biological diversity—Targets—functions. Curr. Top. Med. Chem..

[12] Ferbitz L., Maier T., Patzelt H., Bukau B., Deuerling E., Ban N. (2004). Trigger factor in complex with the ribosome forms a molecular cradle for nascent proteins. Nature.

[13] Rahfeld J.U., Rucknagel K.P., Stoller G., Horne S.M., Schierhorn A., Young K.D., Fischer G. (1996). Isolation and amino acid sequence of a new 22-kDa FKBP-like peptidyl-prolyl cis/trans-isomerase of *Escherichia coli*. Similarity to Mip-like proteins of pathogenic bacteria. J. Biol. Chem..

[14] Saul F.A., Arie J.P., Vulliez-le Normand B., Kahn R., Betton J.M., Bentley G.A. (2004). Structural and functional studies of FkpA from Escherichia coli, a cis/trans peptidyl-prolyl isomerase with chaperone activity. J. Mol. Biol..

[15] Hottenrott S., Schumann T., Plückthun A., Fischer G., Rahfeld J.U. (1997). The Escherichia coli SlyD is a metal ion-regulated peptidyl-prolyl cis/trans-isomerase. J. Biol. Chem..

[16] Weininger U., Haupt C., Schweimer K., Graubner W., Kovermann M., Bruser T., Scholz C., Schaarschmidt P., Žoldák G., Schmid F.X. (2009). NMR solution structure of SlyD from Escherichia coli: Spatial separation of prolyl isomerase and chaperone function. J. Mol. Biol..

[17] Löw C., Neumann P., Tidow H., Weininger U., Haupt C., Friedrich-Epler B., Scholz C., Stubbs M.T., Balbach J. (2010). Crystal structure determination and functional characterization of the metallochaperone SlyD from Thermus thermophilus. J. Mol. Biol..

[18] Van Duyne G.D., Standaert R.F., Karplus P.A., Schreiber S.L., Clardy J. (1991). Atomic structure of FKBP-FK506, an immunophilin-immunosuppressant complex. Science.

[19] Harrison R.K., Stein R.L. (1990). Substrate specificities of the peptidyl prolyl cis-trans isomerase activities of cyclophilin and FK-506 binding protein: Evidence for the existence of a family of distinct enzymes. Biochemistry.

[20] Jakob R.P., Žoldák G., Aumueller T., Schmid F.X. (2009). Chaperone domains convert prolyl isomerases into generic catalysts of protein folding. Proc. Natl. Acad. Sci. USA.

[21] Žoldák G., Aumüller T., Lücke C., Hritz J., Oostenbrink C., Fischer G., Schmid F.X. (2009). A library of fluorescent peptides for exploring the substrate specificities of prolyl isomerases. Biochemistry.

[22] Knappe T.A., Eckert B., Schaarschmidt P., Scholz C., Schmid F.X. (2007). Insertion of a chaperone domain converts FKBP12 into a powerful catalyst of protein folding. J. Mol. Biol..

[23] Jakob R.P., Schmid F.X. (2009). Molecular determinants of a native-state prolyl isomerization. J. Mol. Biol..

[24] Quistgaard E.M., Nordlund P., Löw C. (2012). High-resolution insights into binding of unfolded polypeptides by the PPIase chaperone SlpA. FASEB J. Off. Publ. Fed. Am. Soc. Exp. Biol..

[25] Geitner A.J., Weininger U., Paulsen H., Balbach J., Kovermann M. (2017). Structure-Based Insights into the Dynamics and Function of Two-Domain SlpA from Escherichia coli. Biochemistry.

[26] Suzuki R., Nagata K., Yumoto F., Kawakami M., Nemoto N., Furutani M., Adachi K., Maruyama T., Tanokura M. (2003). Three-dimensional solution structure of an archaeal FKBP with a dual function of peptidyl prolyl cis-trans isomerase and chaperone-like activities. J. Mol. Biol..

[27] Martinez-Hackert E., Hendrickson W.A. (2011). Structural analysis of protein folding by the long-chain archaeal chaperone FKBP26. J. Mol. Biol..

[28] Zhang Z., Li W., Logan T.M., Li M., Marshall A.G. (1997). Human recombinant [C22A] FK506-binding protein amide hydrogen exchange rates from mass spectrometry match and extend those from NMR. Protein Sci. Publ. Protein Soc..

[29] Egan D.A., Logan T.M., Liang H., Matayoshi E., Fesik S.W., Holzman T.F. (1993). Equilibrium Denaturation of Recombinant Human FK Binding Protein in Urea. Biochemistry.

[30] Janowski B., Wöllner S., Schutkowski M., Fischer G. (1997). A protease-free assay for peptidyl-prolyl cis/trans isomerases using standard peptide substrates. Anal. Biochem..

[31] Mücke M., Schmid F.X. (1994). Folding mechanism of ribonuclease T1 in the absence of the disulfide bonds. Biochemistry.

[32] Buchner J., Schmidt M., Fuchs M., Jaenicke R., Rudolph R., Schmid F.X., Kiefhaber T. (1991). GroE Facilitates Refolding of Citrate Synthase by Suppressing Aggregation. Biochemistry.

[33] Scholz C., Eckert B., Hagn F., Schaarschmidt P., Balbach J., Schmid F.X. (2006). SlyD proteins from different species exhibit high prolyl isomerase and chaperone activities. Biochemistry.

[34] Martino L., He Y., Hands-Taylor K.L., Valentine E.R., Kelly G., Giancola C., Conte M.R. (2009). The interaction of the Escherichia coli protein SlyD with nickel ions illuminates the mechanism of regulation of its peptidyl-prolyl isomerase activity. FEBS J..

[35] Skjaerven L., Hollup S.M., Reuter N. (2009). Normal mode analysis for proteins. J. Mol. Struct. THEOCHEM.

[36] Case D.A. (1994). Normal mode analysis of protein dynamics. Curr. Opin. Struct. Biol..

[37] Collinet B., Herve M., Pecorari F., Minard P., Eder O., Desmadril M. (2000). Functionally accepted insertions of proteins within protein domains. J. Biol. Chem..

[38] Ay J., Gotz F., Borriss R., Heinemann U. (1998). Structure and function of the Bacillus hybrid enzyme GluXyn-1: Native-like jellyroll fold preserved after insertion of autonomous globular domain. Proc. Natl. Acad. Sci. USA.

[39] Cherezov V., Rosenbaum D.M., Hanson M.A., Rasmussen S.G., Thian F.S., Kobilka T.S., Choi H.J., Kuhn P., Weis W.I., Kobilka B.K. (2007). High-resolution crystal structure of an engineered human β2-adrenergic G protein–coupled receptor. Science.

[40] Baumlova A., Chalupska D., Róźycki B., Jovic M., Wisniewski E., Klima M., Dubankova A., Kloer D.P., Nencka R., Balla T. (2014). The crystal structure of the phosphatidylinositol 4-kinase II α. EMBO Rep..

[41] Žoldák G., Schmid F.X. (2011). Cooperation of the prolyl isomerase and chaperone activities of the protein folding catalyst SlyD. J. Mol. Biol..

[42] Kovermann M., Schmid F.X., Balbach J. (2013). Molecular function of the prolyl cis/trans isomerase and metallochaperone SlyD. Biol. Chem..

[43] Kovermann M., Zierold R., Haupt C., Löw C., Balbach J. (2011). NMR relaxation unravels interdomain crosstalk of the two domain prolyl isomerase and chaperone SlyD. Biochim. Biophys. Acta.

[44] Kahra D., Kovermann M., Löw C., Hirschfeld V., Haupt C., Balbach J., Hübner C.G. (2011). Conformational plasticity and dynamics in the generic protein folding catalyst SlyD unraveled by single-molecule FRET. J. Mol. Biol..

[45] Kityk R., Vogel M., Schlecht R., Bukau B., Mayer M.P. (2015). Pathways of allosteric regulation in Hsp70 chaperones. Nat. Commun..

[46] Aroul-Selvam R., Hubbard T., Sasidharan R. (2004). Domain insertions in protein structures. J. Mol. Biol..

[47] Ekman D., Bjorklund A.K., Frey-Skott J., Elofsson A. (2005). Multi-domain proteins in the three kingdoms of life: Orphan domains and other unassigned regions. J. Mol. Biol..

[48] Herbst D.A., Jakob R.P., Zahringer F., Maier T. (2016). Mycocerosic acid synthase exemplifies the architecture of reducing polyketide synthases. Nature.

[49] Maier T., Leibundgut M., Ban N. (2008). The crystal structure of a mammalian fatty acid synthase. Science.

[50] Wriggers W., Chakravarty S., Jennings P.A. (2005). Control of protein functional dynamics by peptide linkers. Biopolymers.

[51] Santoro M.M., Bolen D.W. (1988). Unfolding free energy changes determined by the linear extrapolation method. 1. Unfolding of phenylmethanesulfonyl a- chymotrypsin using different denaturants. Biochemistry.

[52] Kabsch W. (2010). Integration, scaling, space-group assignment and post-refinement. Acta Crystallogr. Sect. D Biol. Crystallogr..

[53] Kabsch W. (2010). XDS. Acta Crystallogr. Sect. D Biol. Crystallogr..

[54] Wilson K.P., Yamashita M.M., Sintchak M.D., Rotstein S.H., Murcko M.A., Boger J., Thomson J.A., Fitzgibbon M.J., Black J.R., Navia M.A. (1995). Comparative X-ray structures of the major binding protein for the immunosuppressant FK506 (tacrolimus) in unliganded form and in complex with FK506 and rapamycin. Acta Crystallogr. Sect. D Biol. Crystallogr..

[55] McCoy A.J., Grosse-Kunstleve R.W., Adams P.D., Winn M.D., Storoni L.C., Read R.J. (2007). Phaser crystallographic software. J. Appl. Crystallogr..

[56] Emsley P., Cowtan K. (2004). Coot: Model-building tools for molecular graphics. Acta Crystallogr. Sect. D Biol. Crystallogr..

[57] Adams P.D., Grosse-Kunstleve R.W., Hung L.W., Ioerger T.R., McCoy A.J., Moriarty N.W., Read R.J., Sacchettini J.C., Sauter N.K., Terwilliger T.C. (2002). PHENIX: Building new software for automated crystallographic structure determination. Acta Crystallogr. Sect. D Biol. Crystallogr..

